# A new species of the genus *Quedius* Stephens, 1829, subgenus *Microsaurus* Dejean, 1833, from northeastern North America (Coleoptera, Staphylinidae, Staphylinini, Quediina)

**DOI:** 10.3897/zookeys.126.1647

**Published:** 2011-09-02

**Authors:** Aleš Smetana, Reginald P. Webster

**Affiliations:** 1 Agriculture and Agri-Food Canada, Biodiversity, Central Experimental Farm, K. W. Neatby Bldg., Ottawa, ON K1A 0C6, Canada; 2 Natural Resources Canada, Canadian Forest Service - Atlantic Forestry Centre, 1350 Regent St., P.O. Box 4000, Fredericton, NB, Canada E3B 5P7

**Keywords:** Coleoptera, Staphylinidae, Staphylininae, *Quedius*, subgenus *Microsaurus*, Nearctic, northeastern North America, taxonomy, new species, description, distribution

## Abstract

The paper contains a description of a new species of the subgenus *Microsaurus* Dejean, 1833, of the genus *Quedius* Stephens, 1829, based on specimens from northeastern North America (Canada: Ontario, Quebec, New Brunswick; USA: New Hampshire).

## Introduction

Since the publication of the revision of the tribe Quediini of America north of Mexico by Smetana (1971a), followed by six supplements (Smetana 1971b, 1973, 1976, 1978, 1981, 1990), no additional new species has been described from that territory. The species described as new in this paper has been known to the senior author for many years (specimens from Ontario and New Hampshire) but remained undescribed. Quite recently, after numerous specimens of this species were discovered in New Brunswick and Quebec during 2008–2010, the species attracted new attention. The new species is remarkably similar in general habitus and especially in the coloration to the widely distributed *Quedius erythrogaster* Mannerheim, 1852, but it differs from the latter by several striking character states concerning the chaetotaxy of pronotum and the unique type of punctation of the elytra.

## Material and methods

Acronyms used in the text, when referring to the deposition of the specimens, are as follows:

AFC Natural Resources Canada, Canadian Forest Service - Atlantic Forestry Centre, Fredericton, New Brunswick, Canada.

CNC Canadian National Collection of Insects, Agriculture and Agri-Food Canada, Ottawa, Canada.

RWC Reginald P. Webster collection, Charters Settlement, New Brunswick, Canada.

UNHC University of New Hampshire collection, Durham, New Hampshire, United States.

The features on the aedoeagus are described as seen in ventral view (with paramere up).

The locality data are given exactly as they appear on the locality labels. The measurement ratios given in the description are average values. Specimens were photographed using an image processing system (Nikon SMZ 1500 stereoscopic microscope: Nikon digital camera DXM 1200F; and Adobe Photoshop software).

## Taxonomy

### Quedius (Microsaurus) bicoloris sp. n.

urn:lsid:zoobank.org:act:2D27D89F-7565-42CD-A065-2A790E8E1213

http://species-id.net/wiki/Quedius_(Microsaurus)_bicoloris

Figs 1, 2 5–8

#### Type locality.

 Canada, New Brunswick, York Co. 15 km W of Tracy, off Rt. 645, 45.6848°N, 66.8821°W.

#### Type material.

 Holotype (male): CAN., NEW BRUNSWICK, YORK CO. 15 km W of Tracy, off Rt. 645, 45.6848°N, 66.8821°W 25 April–4 May 2009 R. Webster & M.-A. Giguère. coll. // red pine forest Lindgren funnel trap // PHOTO 10- 006 P. Sylvestre”. In CNC, Ottawa. Allotype (female): same labels as holotype, - last label + AFCF0005479. In CNC, Ottawa.

Paratypes: **CANADA:** NEW BRUNSWICK: same labels as allotype, but date 4–11 May 2009 + AFCF0005476, 1 ♀ (AFC); same labels as allotype, but date 19–25 May 2009 + AFC F0005480, 1 ♀ (AFC); same labels as holotype (except for last label), 1 ♀ (RWC); same labels as holotype (except for last label) but date 25 May–1 June 2009, 1 ♂ (CNC); same labels as holotype (except for last label) but date 19–25 May 2009 + AFCF0005481, 1 ♀ (AFC); CAN., NEW BRUNSWICK, YORK CO., 14 km WSW of Tracy, S of Rt. 645, 45.6741°N, 66.8661°W 26 April–10 May 2010, R. Webster & C. MacKay. coll. // Old mixed forest with Red and White Spruce, Red and White Pine, Balsam Fir. Eastern White Cedar. Red Maple and *Populus* sp. Lindgren funnel trap, 1 ♀ (RWC); same labels as previous + AFCF 0005484, 1 ♂ (CNC); same labels as previous + AFCF0005483, 1 ♂ (AFC). CARLETON CO., Jackson Falls., “Bell Forest” 46.2200°N, 67.7231°W, 9–14 May 2009, R. Webster & M.-A. Giguère. coll. // Rich Appalachian hardwood forest with some conifers, Lindgren funnel trap , 1 ♀ (CNC); same two labels but date 23–28 April 2010, 1 ♂, 1 ♀ (RWC); same two labels as previous but date 14–20 May, 1 ♂ (CNC); same two labels as previous but date 20–26 May 2009 + Staph. Species 544, 1 ♀ (RWC); same two labels as previous but date 16–21 June 2009, 1♀ (RWC); same two labels as previous but date 12–19 June 2010 and R. P. Webster coll., 1 ♂ (RWC); same two labels as previous but date 19–27 May 2010, 1 ♂, 1 ♀ (CNC, RWC). QUEENS CO., Cranberry Lake P. N. A. 46.1125°N, 65.6075°W 24 April–5 May 2009 R. Webster & M.-A. Giguère. coll. // Red oak forest Lindgren funnel trap // AFCF 0005475, 1 ♂ (AFC); same two labels as previous but date 27 May–5 June 2009 + AFCF 0005477, 1 ♂ (AFC); same two labels as previous but date 18–25 June 2009 + AFCF 0005476, 1 ♀ (AFC). SUNBURY CO., Acadia Research Forest 45.9866N 66.3841W 28 April–8 May 2009, R. Webster & M.-A. Giguêre, coll. // Red spruce forest with red maple and balsam fir Lindgren funnel trap // AFCF 0005482, 1 ♀ (AFC). ONTARIO: ON, Constance Bay, 1. X. 1953, EC Becker, 1 ♂, 1 ♀ (CNC). QUEBEC: CANADA, Qc . Co Gatineau Buckingham 45°34'N, 75°28'W, 12.-19. VI. 2000 Project Verglas 2000 // Lindgren Érablière á sucre 2000-3-0680 // *Quedius* sp. 1 Dét. G. Pelletier 2002, 1 ♀ (CNC). **UNITED STATES:** NEW HAMPSHIRE: Strafford Co., Durham Foss Fm. Rd. Water Tower X-22-1980, Coll. W. J. Morse, 1 ♂ (UNHC); USA: NH: Straf. Co., 1 mi N Durham, water tower 10-30-1982, W. J. Morse, 1 ♂ (UNHC).

#### Diagnosis.

**Figures 1–4.**
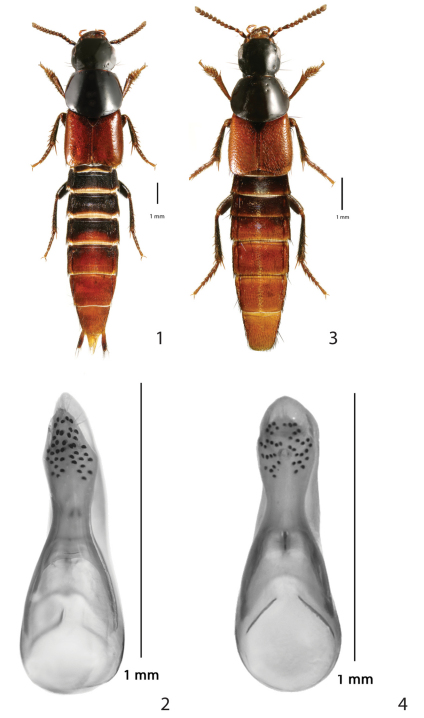
*Quedius bicoloris* sp. n.: **1** habitus **2** aedoeagus, ventral view. *Quedius erythrogaster* Mannerheim, 1852: **3** habitus **4** aedoeagus, ventral view.

*Quedius bicoloris* is in general habitus and coloration quite similar to *Quedius erythrogaster*Mannerheim, 1852, but differs in several external characters, as well as in the differently shaped aedoeagus (Figs 1–4). The main diagnostic external charactersare the reduction of each of the dorsal rows on the pronotum to one puncture situated close to the anterior margin of pronotum, and the unique character of the elytral punctation (see the description). The aedoeagus, although it is of the same general build, is markedly different, both in the shape of the apical portion of median lobe and the shape of the paramere (Figs 2, 4). Tergite 10 of the female genital segment is also different (Figs 8, 11).

#### Description.

 Head, pronotum, and scutellum black. Elytra rusty red. First two and basal half of third visible abdominal tergites or first three visible tergites entirely piceous black to black, remainder of abdominal tergites rusty red to pale reddish. Mandibles piceous black to black, maxillary and labial palpi testaceous. Antennae piceous, becoming gradually variably paler toward apex. Legs piceous, with dorsal faces of front tibiae and all tarsi variably paler. Head of rounded quadrangular shape, wider than long (ratio 1.21), usually slightly widened behind eyes, posterior angles obsolete; eyes rather small, feebly convex, tempora somewhat longer than eyes seen from above (ratio 1.20); no additional setiferous punctures between anterior frontal punctures; posterior frontal puncture shifted markedly posteriad, situated close to posterior margin of head, two punctures between it and posterior margin of head (one of these punctures missing unilaterally in some specimens); temporal puncture shifted posteriad, separated from posteriomedial margin of eye by distance about twice as long as its distance from posterior margin of head; surface of head with very fine, very dense microsculpture of transverse and oblique waves, with intermixed fine micropunctulae that become gradually coarser toward posterior portions of head. Antennae short, moderately widened toward apex, segments 2 and 3 subequal in length, segments 4 and 5 about as long as wide, segments 6 to 10 wider than long, gradually becoming shorter, with segments 9 and 10 markedly transverse, last segment about as long as two preceding segments combined. Pronotum wider than long (ratio 1.15), widest at about posterior third, narrowed anteriad, with lateral margins continuously arcuate with broadly rounded base, transversely convex, lateral portions not explanate; dorsal rows each with only one puncture at anterior pronotal margin (puncture occasionally doubled unilaterally); sublateral rows each with one puncture close to anterior margin of pronotum; microsculpture similar to that on head, but slightly denser, intermixed micropunctulae quite fine. Scutellum impunctate, surface with microsculpture of very fine waves. Elytra moderately long, at base narrower than pronotum at widest point, no more than vaguely dilated posteriad, at suture as long as, at sides somewhat longer than pronotum at midline (ratio 1.14); punctation dual, consisting of moderately coarse and very fine punctures; coarser punctures on each elytron forming a group on medial half of elytral base laterad of scutellum and from there extending in a sparse, very variable, unstable erratic pattern posteriad toward posterior margin of each elytron; irregular row of coarser punctures present along suture of each elytron and on margin of elytra; very fine punctures present in irregular, variable pattern on entire surface of each elytron, including lateral portion; surface between punctures without appreciable microsculpture. Wings fully developed. Abdomen with tergite 7 (fifth visible) with fine whitish apical seam of palisade setae; tergite 2 (in front of fully visible tergite 3) impunctate (some micropunctulae present); punctation of abdominal tergites dense at base of each tergite, becoming sparser toward apex of each tergite, and in general toward apex of abdomen; pubescence piceous; surface between punctures with exceedingly fine microsculpture of broken striae.

**Figures 5–11.**
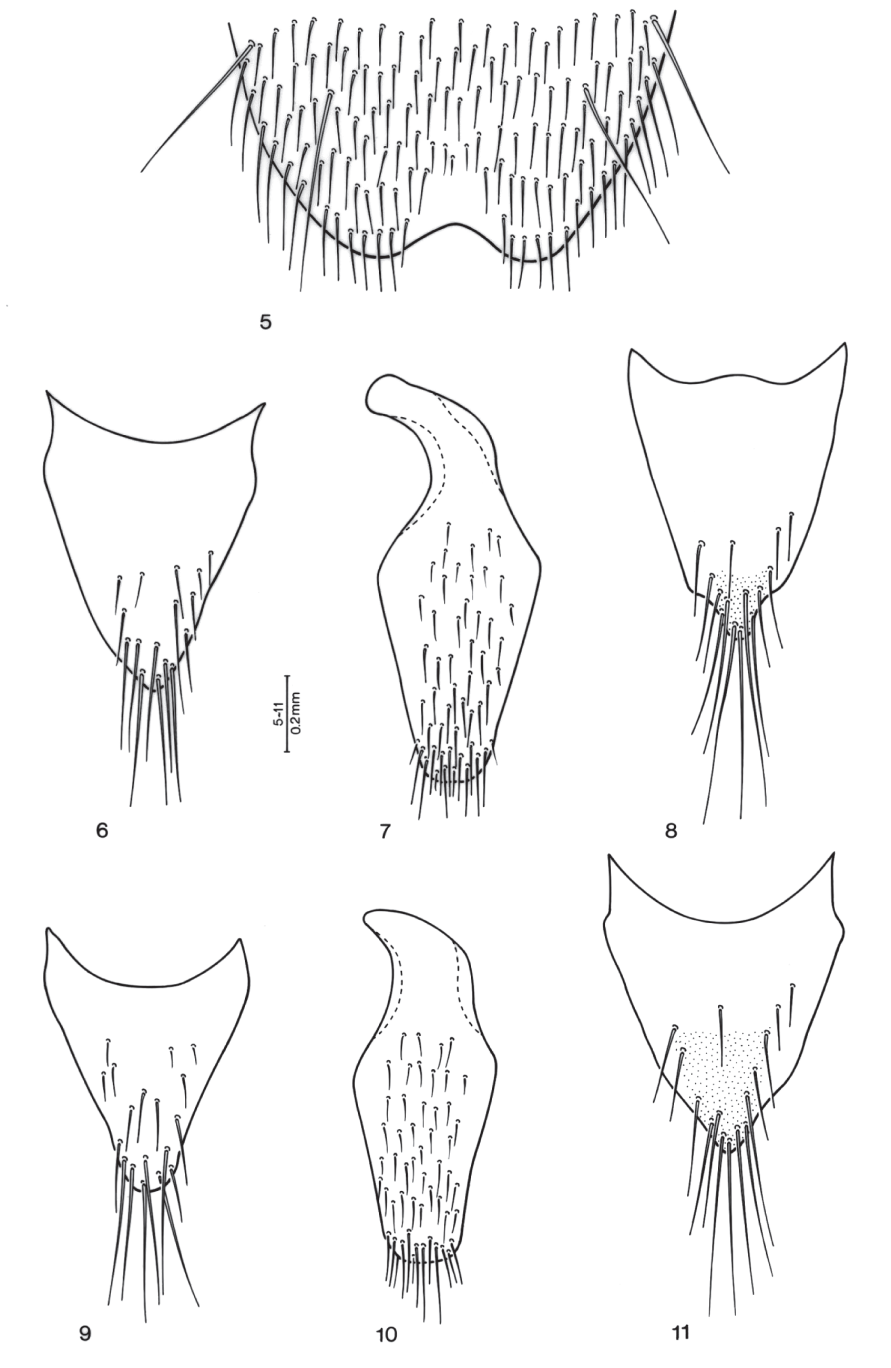
*Quedius bicoloris* sp. n.: **5** apical portion of male sternite 8 **6** tergite 10 of male genital segment **7** sternite 9 of male genital segment **8** tergite 3–10 of female genital segment. *Quedius erythrogaster* Mannerheim, 1852: **9** tergite 10 of male genital segment **10** sternite 9 of male genital segment **11** tergite 10 of female genital segment.

#### Male.

 First four segments of front tarsus moderately dilated, sub-bilobed, each densely covered with tenent setae ventrally; segment 2 about as wide as apex of tibia; segment 4 narrower than preceding segments. Sternite 8 with two macrosetae on each side, apical margin with moderately wide and rather shallow, obtusely arcuate medioapical emargination, small triangular area before emargination flattened and smooth (Fig. 5); sternite 7 with apical margin minutely, arcuately emarginate medially; sternite 6 with a small field of denser setae mediobasally. Genital segment with tergite 10 markedly, evenly narrowed toward narrowly arcuate apex, setose at and around apex, otherwise with only sparse, minute setae (Fig. 6); sternite 9 with moderately robust basal portion, apical portion arcuate apically, without differentiated setae, setose as in Fig. 7. Aedoeagus (Fig. 2) rather robust; median lobe subparallelsided in middle portion, anteriorly narrowed into short apical portion with narrowly arcuate apex; paramere short, robust, apical portion of characteristic diamond shape, apex of apical portion distinctly not reaching apex of median lobe; four setae at apex and two similar setae at each lateral margin below apex; sensory peg setae on underside of paramere quite numerous, covering entire apical portion with exception of small triangular mediobasal area.

#### Female.

 First four segments of front tarsus similar to those of male, but markedly less dilated, segment 2 slightly narrower than apex of tibia (ratio 0.90). Tergite 10 of genital segment similar to that of *Quedius erythrogaster*, but smaller and narrower, with pigmented medioapical area much smaller (Figs 8, 11).

Length 7.5–9.5 mm.

#### Geographical distribution.

**Map 1.**
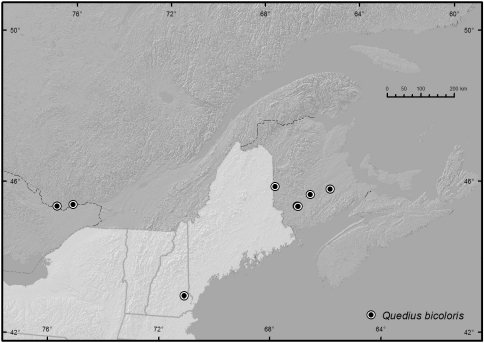
Presently known distribution of *Quedius bicoloris*.

*Quedius bicoloris* is distributed in northeastern North America (Map 1). It is presently known from New Brunswick, Ontario, and Quebec in Canada and, so far, only from New Hampshire in the United States. It is expected to be more widely distributed.

#### Bionomics.

Little is known about biology of the species. The Quebec specimen was taken from a Lindgren funnel trap in a sugar maple stand, the specimens from New Hampshire were all taken sitting on cool walls of a water tower. The New Brunswick specimens were collected from Lindgren funnel traps during a study to develop a general attractant for the detection of invasive species of Cerambycidae. Adults were taken in a variety of forest types, including an old (120-180-year-old trees) red pine forest, old mixed forest with red and white spruce, red and white pine, balsam fir, eastern white cedar, red maple, and *Populus* sp., Rich Appalachian Hardwood Forest (sugar maple, beech, ash, butternut), and a red oak forest, red spruce forest with red maple and balsam fir. These traps mimic tree trunks and are often effective for sampling species of Coleoptera that live in microhabitats associated with standing trees (Lindgren 1983). This species is probably associated with standing trees, possibly living in subcortical habitats or in fungi on trees.

#### Etymology.

The specific epithet is the combination of Latin adverb *bis* (twice) and the genitive of the noun *color*, -*oris*, m (meaning of two colors). To be treated as noun in apposition. It refers to the coloration of the body of the species.

## Supplementary Material

XML Treatment for Quedius (Microsaurus) bicoloris
